# (+)-Methyl 3β-acet­oxy-13-carb­oxy-19-hy­droxy-11-oxo-*C*-norolean-18-en-30-oate γ-lactone

**DOI:** 10.1107/S1600536810036901

**Published:** 2010-09-18

**Authors:** Rawindra Gaware, Laszlo Czollner, Ulrich Jordis, Kurt Mereiter

**Affiliations:** a Institute of Applied Synthetic Chemistry, Vienna University of Technology, Getreidemarkt 9/163, A-1060 Vienna, Austria; b Institute of Chemical Technologies and Analytics, Vienna University of Technology, Getreidemarkt 9/164SC, A-1060 Vienna, Austria

## Abstract

The title compound, C_33_H_46_O_7_, is an unusual oxydation product of the therapeutic agent glycyrrhetinic acid that has, in comparison to the latter, a distinctly altered triterpene structure with one five- and four six-membered carbocycles complemented by a γ-lactone ring with a spiro-junction and a ring double bond. The junction between the five-membered ring *C*, a cyclo­penta­none ring, and the six-membered ring *D*, previously in question, was found to be *cis*, confirming earlier structure assignments based solely on chemical transformations. In the solid state, the compound exhibits five intra- and four inter­molecular C—H⋯O inter­actions with H⋯O distances less than or equal to 2.70 Å and C—H⋯O greater than 100°.

## Related literature

For the synthesis and structure elucidation of the title compound by chemical methods, see: Brownlie & Spring (1956 ▶); Jeger *et al.* (1944 ▶). For overviews of the therapeutic aspects of the parent compounds glycyrrhetinic acid and glycyrrhizin, see: Asl & Hosseinzadeh (2008 ▶); Baran *et al.* (1974 ▶); Kitagawa (2002 ▶). For recent research on the synthesis of new derivatives of glycyrrhetinic acid and their medicinal potency, see: Classen-Houben *et al.* (2009 ▶); Beseda *et al.* (2010 ▶); Amer *et al.* (2010 ▶).
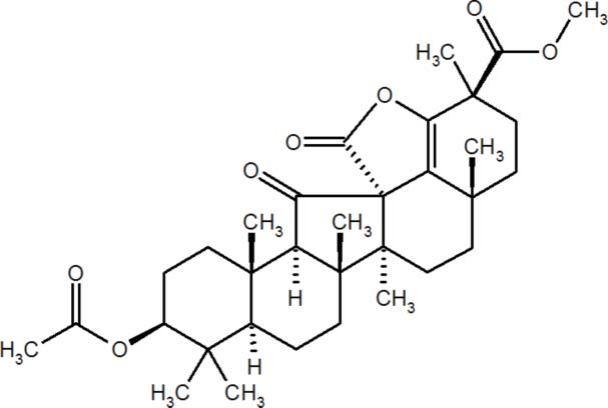


## Experimental

### 

#### Crystal data

C_33_H_46_O_7_
*M* *_r_* = 554.70
Orthorhombic, 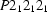

*a* = 7.6310 (4) Å
*b* = 16.7922 (9) Å
*c* = 22.3822 (13) Å
*V* = 2868.1 (3) Å^3^
*Z* = 4
Mo *K*α radiation
μ = 0.09 mm^−1^
*T* = 100 K
0.56 × 0.44 × 0.32 mm

#### Data collection

Bruker Kappa APEXII CCD diffractometer
Absorption correction: multi-scan (*SADABS*; Bruker, 2008 ▶) *T*_min_ = 0.88, *T*_max_ = 0.97
41398 measured reflections
4654 independent reflections
4540 reflections with *I* > 2σ(*I*)
*R*_int_ = 0.026

#### Refinement

*R*[*F* ^2^ > 2σ(*F* ^2^)] = 0.030
*wR*(*F* ^2^) = 0.080
*S* = 1.05
4654 reflections
370 parameters
H-atom parameters constrained
Δρ_max_ = 0.34 e Å^−3^
Δρ_min_ = −0.19 e Å^−3^

### 

Data collection: *APEX2* (Bruker, 2008 ▶); cell refinement: *SAINT* (Bruker, 2008 ▶); data reduction: *SAINT*, *SADABS* and *XPREP* (Bruker, 2008 ▶); program(s) used to solve structure: *SHELXS97* (Sheldrick, 2008 ▶); program(s) used to refine structure: *SHELXL97* (Sheldrick, 2008 ▶); molecular graphics: *SHELXTL* (Sheldrick, 2008 ▶) and *Mercury* (Macrae *et al.*, 2006 ▶); software used to prepare material for publication: *PLATON* (Spek, 2009 ▶) and *publCIF* (Westrip, 2010 ▶).

## Supplementary Material

Crystal structure: contains datablocks I, global. DOI: 10.1107/S1600536810036901/bt5349sup1.cif

Structure factors: contains datablocks I. DOI: 10.1107/S1600536810036901/bt5349Isup2.hkl

Additional supplementary materials:  crystallographic information; 3D view; checkCIF report

## Figures and Tables

**Table 1:** Hydrogen-bond geometry (Å, °)

*D*—H⋯*A*	*D*—H	H⋯*A*	*D*⋯*A*	*D*—H⋯*A*
C2—H2*B*⋯O5	0.99	2.58	3.1121 (14)	114
C9—H9⋯O6	1.00	2.43	3.1601 (13)	129
C23—H23*A*⋯O1	0.98	2.49	2.9084 (15)	106
C25—H25*A*⋯O2	0.98	2.60	3.2898 (16)	127
C27—H27*B*⋯O6	0.98	2.49	3.0454 (16)	116
C1—H1*A*⋯O5^i^	0.99	2.63	3.4839 (15)	145
C5—H5⋯O5^ii^	1.00	2.64	3.5313 (14)	149
C7—H7*B*⋯O2^iii^	0.99	2.59	3.1743 (15)	118
C7—H7*B*⋯O5^ii^	0.99	2.70	3.5497 (15)	145

Symmetry codes: (i) 
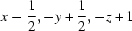
; (ii) 
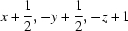
; (iii) 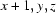.

**Fig. 1.**
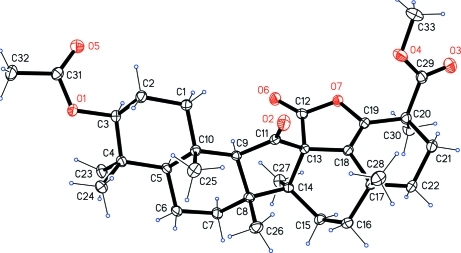
The molecular structure of (I) with displacement ellipsoids for the non-hydrogen atoms drawn at the 50% probability level.

**Fig. 2.**
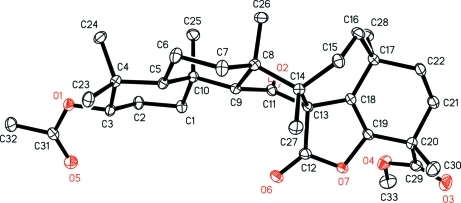
The molecular structure of (I) in a side-view showing the conformation of the rings more clearly. Hydrogen atoms omitted for clarity.

**Fig. 3.**
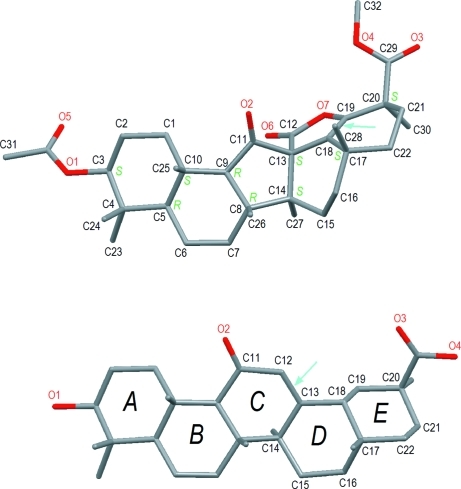
Molecular structure of (I) (top) in comparison with the parent compound glycyrrhetinic acid (bottom) in its DMSO solvate (Beseda et al., 2010). Hydrogen atoms omitted for clarity, ring designation in large italics (only for bottom molecule), configuration of the asymmetric carbon atoms green (I), blue arrows indicate the C=C double bonds. Atom numeration in (I) follows a widely accepted designation in glycyrrhetinic acid.

**Fig. 4.**
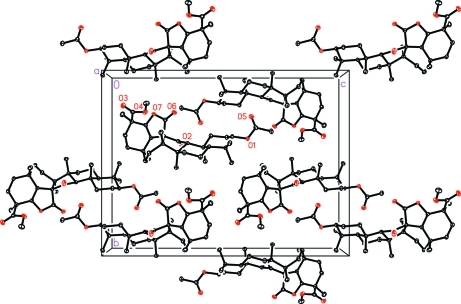
Packing diagram of (I) in a view down the a axis. Hydrogen atoms omitted for clarity.

## supplementary crystallographic information

### Comment

Glycyrrhetinic acid (18β-glycyrrhetinic acid, GA), a pentacyclic triterpenoid, is the aglycone of glycyrrhizin, the main sweet tasting compound from liquorice root that is in use as flavoring and sweetener in candies and food (Kitagawa, 2002). This compound has a long record as therapeutic agent with antiinflammatory, antiulcer, antiallergic, and other activity by being active towards 11β-hydroxysteroid dehydrogenases and consequently modulating the steroid hormone cortisol (Baran *et al.*, 1974; Asl & Hosseinzadeh, 2008). With these features glycyrrhetinic acid is of ongoing interest for creating new derivatives aimed at improving or diversifying its therapeutical profile.

In context with corresponding research (Classen-Houben *et al.*, 2009; Beseda *et al.*, 2010; Amer *et al.*, 2010) we came across the title compound, (I), which was of interest because it is an unusual derivative of glycyrrhetinic acid. Obtained initially by the Nobel laureate Ruzicka and his group (Jeger *et al.*, 1944), it was later on synthesized from methyl glycyrrhetate acetate, C_33_H_50_O_5_, in a one-step reaction by oxydation with SeO_2_ under introduction of two more oxygen atoms, elimination of four hydrogen atoms, rearrangement of one ring, and generation of an additional unsaturated γ-lactone ring (Brownlie & Spring, 1956). The assignment of a structural formula to this compound was as yet based on combustion analysis, derivatizations, chemical tests for funtional groups and stereochemical considerations but not on present day methods like NMR spectroscopy. Therefore the structural formula of (I) was partly open to question and it was considered worth to secure it by X-ray crystallography.

The result of the present work is shown in Figures 1 and 2. In order to facilitate the discussion, a comparison of the molecular structure of (I) with the parent compound 18β-glycyrrhetinic acid (GA) is given in Fig. 3. This figure includes also the chiralities of the nine asymmetric carbon atoms in (I), which prove that the assignment of Brownlie & Spring (1956) is correct, which is of relevance for carbon C13 (crystallographic atom numbering). In both compounds, (I) and GA, are the six membered rings A and B adopting the usual chair conformation and having a *trans*-link. On transition from GA to (I), the cyclohex-2-enone ring C is oxydatively cleaved between C11 and C12 and transforms into a cyclopentanone ring, whereas the former sixth ring carbon, C12, is utilized to generate a new γ-lactone ring with a new double bond between C18 and C19. By this transformation the carbon C13, initially of *sp*^2^-type, becomes *sp*^3^-type and chiral with *S*-configuration, as proven by X-ray diffraction. Hence, the junction between rings C and D in (I) is of a *cis*-type. Compared with GA the introduction of the new γ-lactone ring and the double bond C18—C19 in (I) changes the conformation of rings D and E drastically, namely from chair and chair in GA to twist-boat and twisted half-chair in (I). Therefore the shape of the molecule in (I) is singificantly altered in comparison to GA and ordinary derivatives thereof (Beseda *et al.*, 2010). Bond lengths and angles in (I), listed below are largely normal except for the two most congested carbon atoms C13 and C14 (both *sp*^3^), which show two notably elongated bonds (C13—C14 = 1.5910 (15) Å, C14—C8 = 1.6026 (15) Å) and two unusually large bond angles (C11—C13—C18 = 119.9 (1) ° and C8—C14—C15 = 115.4 (1) °). The double bond C18—C19 shared by rings E and F measures 1.3299 (15) Å. A packing diagram of (I) is shown in Fig. 4. Apart from five intramolecular C—H···O interactions there are only four intermolecular C—H···O interactions with H···O ≤ 2.70 Å and C—H···O > 100° (Table 1) and the molecules are therefore held together mainly by van der Waals interactions.

### Experimental

The title compound was synthesized similar to the method of Brownlie & Spring (1956). To a solution of methyl glycyrrhetate acetate (500 mg, 0.98 mmol) in glacial acetic acid (30 ml) was added selenium dioxide (500 mg, 4.51 mmol) and the mixture stirred at 120 °C oil bath temperature. After 24 h the solvent was removed under vacuum, the residue diluted with water (100 ml) and extracted with dichloromethane (3 × 40 ml). The combined organic phase was washed with brine (10 ml) and dried over MgSO_4_. The drying agent was removed by filtration, and the filtrate transferred to a round-bottom flask. The solution was evaporated to a constant weight with a rotary evaporator to leave 180 mg (36%) of pale-yellow material. An analytical sample was obtained by recrystallization from ethanol and melted at 291–292 °C. ^1^H NMR (200 MHz, CDCl_3_): δ 4.46 (m,1*H*), 3.72 (s, 3H), 3.07 (s, 1H), 2.60 (m, 1H), 2.04 (s, 3H), 2.18–0.80 (m, 19H),1.42 (s, 3H), 1.182 (s, 3H), 1.15 (s, 6H), 1.09 (s, 3H), 0.88 (s, 3H). ^13^C NMR (50 MHz, CDCl_3_): δ 203.8, 174.6, 174.3, 170.7, 151.4, 119.1, 80.3, 67.6, 65.5, 55.1, 52.7, 48.3,43.2, 42.3, 37.7, 36.6, 36.3, 35.6, 35.1, 33.9, 32.6, 31.2, 30.6, 28.0, 26.7,26.6, 23.2, 23.0, 21.2, 21.0, 18.8, 17.1, 16.3.

### Refinement

All H atoms were placed in calculated positions and thereafter treated as riding. A torsional parameter was refined for each methyl group. *U*_iso_(H) = 1.2*U*_eq_(C_non-methyl_) and *U*_iso_(H) = 1.5*U*_eq_(C_methyl_) were used. Prior to final refinement the 3554 Friedel pairs were merged.

### Crystal data

**Table:**

C_33_H_46_O_7_	*D*_x_ = 1.285 Mg m^−^^3^
*M**_r_* = 554.70	Melting point: 565 K
Orthorhombic, *P*2_1_2_1_2_1_	Mo *K*α radiation, λ = 0.71073 Å
Hall symbol: P 2ac 2ab	Cell parameters from 9893 reflections
*a* = 7.6310 (4) Å	θ = 2.6–30.5°
*b* = 16.7922 (9) Å	µ = 0.09 mm^−^^1^
*c* = 22.3822 (13) Å	*T* = 100 K
*V* = 2868.1 (3) Å^3^	Prism, colourless
*Z* = 4	0.56 × 0.44 × 0.32 mm
*F*(000) = 1200	

### Data collection

**Table:**

Bruker Kappa APEXII CCD diffractometer	4654 independent reflections
Radiation source: fine-focus sealed tube	4540 reflections with *I* > 2σ(*I*)
graphite	*R*_int_ = 0.026
φ and ω scans	θ_max_ = 30.0°, θ_min_ = 2.9°
Absorption correction: multi-scan (*SADABS*; Bruker, 2008)	*h* = −10→10
*T*_min_ = 0.88, *T*_max_ = 0.97	*k* = −23→22
41398 measured reflections	*l* = −30→31

### Refinement

**Table:**

Refinement on *F*^2^	Primary atom site location: structure-invariant direct methods
Least-squares matrix: full	Secondary atom site location: difference Fourier map
*R*[*F*^2^ > 2σ(*F*^2^)] = 0.030	Hydrogen site location: inferred from neighbouring sites
*wR*(*F*^2^) = 0.080	H-atom parameters constrained
*S* = 1.05	*w* = 1/[σ^2^(*F*_o_^2^) + (0.0571*P*)^2^ + 0.3708*P*] where *P* = (*F*_o_^2^ + 2*F*_c_^2^)/3
4654 reflections	(Δ/σ)_max_ < 0.001
370 parameters	Δρ_max_ = 0.34 e Å^−^^3^
0 restraints	Δρ_min_ = −0.19 e Å^−^^3^

### Special details

**Table:**

Geometry. All e.s.d.'s are estimated using the full covariance matrix. The cell e.s.d.'s are taken into account individually in the estimation of e.s.d.'s in distances, angles and torsion angles; correlations between e.s.d.'s in cell parameters are only used when they are defined by crystal symmetry.
Refinement. Refinement of *F*^2^ against ALL reflections. The weighted *R*-factor *wR* and goodness of fit *S* are based on *F*^2^, conventional *R*-factors *R* are based on *F*, with *F* set to zero for negative *F*^2^. The threshold expression of *F*^2^ > σ(*F*^2^) is used only for calculating *R*-factors(gt) *etc*. and is not relevant to the choice of reflections for refinement. *R*-factors based on *F*^2^ are statistically about twice as large as those based on *F*, and *R*- factors based on ALL data will be even larger.

### Fractional atomic coordinates and isotropic or equivalent isotropic displacement parameters (Å^2^)

**Table:**

	*x*	*y*	*z*	*U*_iso_*/*U*_eq_	
O1	0.58647 (12)	0.36304 (5)	0.59280 (3)	0.01542 (16)	
O2	0.21014 (12)	0.38578 (6)	0.30563 (4)	0.02061 (18)	
O3	0.07765 (16)	0.16391 (6)	0.05217 (4)	0.0278 (2)	
O4	0.02940 (13)	0.20027 (5)	0.14712 (4)	0.02104 (18)	
O5	0.49099 (13)	0.23786 (5)	0.60940 (4)	0.02102 (18)	
O6	0.51640 (14)	0.21478 (5)	0.28433 (4)	0.02095 (19)	
O7	0.38773 (12)	0.22126 (5)	0.19472 (4)	0.01530 (16)	
C1	0.38579 (15)	0.34494 (7)	0.43805 (5)	0.01347 (19)	
H1A	0.2624	0.3473	0.4245	0.016*	
H1B	0.4337	0.2923	0.4267	0.016*	
C2	0.39189 (15)	0.35369 (7)	0.50625 (5)	0.0144 (2)	
H2A	0.3375	0.4049	0.5180	0.017*	
H2B	0.3242	0.3100	0.5250	0.017*	
C3	0.57997 (15)	0.35113 (7)	0.52821 (5)	0.01303 (19)	
H3	0.6294	0.2974	0.5190	0.016*	
C4	0.70187 (15)	0.41476 (6)	0.50091 (5)	0.01278 (19)	
C5	0.68261 (15)	0.40901 (6)	0.43176 (5)	0.01175 (19)	
H5	0.7267	0.3546	0.4217	0.014*	
C6	0.80504 (16)	0.46575 (7)	0.39755 (5)	0.0160 (2)	
H6A	0.9228	0.4647	0.4161	0.019*	
H6B	0.7594	0.5208	0.4005	0.019*	
C7	0.82026 (15)	0.44215 (7)	0.33124 (5)	0.0155 (2)	
H7A	0.8955	0.4812	0.3103	0.019*	
H7B	0.8769	0.3893	0.3282	0.019*	
C8	0.64014 (15)	0.43912 (6)	0.30072 (5)	0.01179 (18)	
C9	0.51682 (14)	0.38750 (6)	0.34039 (4)	0.01017 (18)	
H9	0.5757	0.3343	0.3420	0.012*	
C10	0.49178 (14)	0.41083 (6)	0.40651 (5)	0.01149 (18)	
C11	0.36470 (15)	0.37522 (6)	0.29852 (5)	0.01211 (19)	
C12	0.46147 (16)	0.25521 (6)	0.24456 (5)	0.0144 (2)	
C13	0.45088 (15)	0.34696 (6)	0.23907 (5)	0.01111 (18)	
C14	0.63502 (14)	0.39140 (6)	0.23874 (5)	0.01162 (18)	
C15	0.64754 (15)	0.44362 (7)	0.18159 (5)	0.0145 (2)	
H15A	0.7407	0.4837	0.1881	0.017*	
H15B	0.6864	0.4090	0.1483	0.017*	
C16	0.48210 (16)	0.48775 (7)	0.16123 (5)	0.0156 (2)	
H16A	0.5100	0.5191	0.1250	0.019*	
H16B	0.4464	0.5256	0.1929	0.019*	
C17	0.32639 (15)	0.43173 (6)	0.14723 (5)	0.01265 (19)	
C18	0.35306 (14)	0.35531 (6)	0.18125 (5)	0.01168 (19)	
C19	0.32842 (15)	0.28331 (6)	0.15797 (5)	0.01295 (19)	
C20	0.27038 (16)	0.26123 (7)	0.09642 (5)	0.0144 (2)	
C21	0.20702 (17)	0.33903 (7)	0.06549 (5)	0.0171 (2)	
H21A	0.2051	0.3309	0.0217	0.021*	
H21B	0.0859	0.3510	0.0786	0.021*	
C22	0.32516 (17)	0.41004 (7)	0.08013 (5)	0.0157 (2)	
H22A	0.2851	0.4568	0.0569	0.019*	
H22B	0.4463	0.3976	0.0674	0.019*	
C23	0.89158 (16)	0.39174 (7)	0.51797 (5)	0.0168 (2)	
H23A	0.8966	0.3786	0.5606	0.025*	
H23B	0.9701	0.4366	0.5097	0.025*	
H23C	0.9283	0.3454	0.4944	0.025*	
C24	0.66240 (17)	0.49816 (7)	0.52613 (5)	0.0168 (2)	
H24A	0.6884	0.4991	0.5690	0.025*	
H24B	0.5384	0.5108	0.5198	0.025*	
H24C	0.7353	0.5377	0.5056	0.025*	
C25	0.39079 (17)	0.48996 (7)	0.41380 (5)	0.0171 (2)	
H25A	0.3027	0.4945	0.3821	0.026*	
H25B	0.4729	0.5347	0.4110	0.026*	
H25C	0.3327	0.4909	0.4528	0.026*	
C26	0.57387 (17)	0.52513 (6)	0.29370 (5)	0.0169 (2)	
H26A	0.5942	0.5545	0.3309	0.025*	
H26B	0.4482	0.5246	0.2848	0.025*	
H26C	0.6370	0.5511	0.2610	0.025*	
C27	0.78590 (16)	0.33104 (7)	0.23470 (5)	0.0165 (2)	
H27A	0.7713	0.2983	0.1988	0.025*	
H27B	0.7849	0.2968	0.2702	0.025*	
H27C	0.8978	0.3596	0.2326	0.025*	
C28	0.15441 (17)	0.47305 (7)	0.16436 (6)	0.0181 (2)	
H28A	0.1514	0.4816	0.2077	0.027*	
H28B	0.0552	0.4395	0.1525	0.027*	
H28C	0.1467	0.5245	0.1438	0.027*	
C29	0.11726 (16)	0.20249 (7)	0.09537 (5)	0.0166 (2)	
C30	0.42425 (19)	0.22355 (8)	0.06203 (6)	0.0219 (2)	
H30A	0.4667	0.1767	0.0838	0.033*	
H30B	0.5193	0.2625	0.0583	0.033*	
H30C	0.3847	0.2075	0.0222	0.033*	
C31	0.54224 (16)	0.30102 (7)	0.62791 (5)	0.0152 (2)	
C32	0.5655 (2)	0.32204 (8)	0.69276 (5)	0.0236 (3)	
H32A	0.5682	0.2732	0.7167	0.035*	
H32B	0.4675	0.3555	0.7059	0.035*	
H32C	0.6758	0.3511	0.6980	0.035*	
C33	−0.11701 (18)	0.14577 (8)	0.14807 (7)	0.0252 (3)	
H33A	−0.1760	0.1490	0.1869	0.038*	
H33B	−0.0746	0.0914	0.1416	0.038*	
H33C	−0.1998	0.1600	0.1163	0.038*	

### Atomic displacement parameters (Å^2^)

**Table:**

	*U*^11^	*U*^22^	*U*^33^	*U*^12^	*U*^13^	*U*^23^
O1	0.0208 (4)	0.0152 (3)	0.0103 (3)	−0.0016 (3)	−0.0006 (3)	−0.0002 (3)
O2	0.0112 (4)	0.0337 (5)	0.0169 (4)	0.0005 (4)	0.0006 (3)	0.0015 (4)
O3	0.0385 (6)	0.0258 (5)	0.0192 (4)	−0.0091 (5)	−0.0060 (4)	−0.0045 (3)
O4	0.0193 (4)	0.0213 (4)	0.0225 (4)	−0.0068 (4)	0.0010 (4)	−0.0053 (3)
O5	0.0262 (5)	0.0170 (4)	0.0199 (4)	−0.0032 (4)	−0.0017 (4)	0.0010 (3)
O6	0.0302 (5)	0.0131 (4)	0.0195 (4)	−0.0009 (4)	−0.0085 (4)	0.0036 (3)
O7	0.0206 (4)	0.0104 (3)	0.0149 (3)	0.0002 (3)	−0.0048 (3)	0.0009 (3)
C1	0.0123 (4)	0.0160 (5)	0.0121 (4)	−0.0032 (4)	0.0002 (4)	0.0012 (4)
C2	0.0129 (5)	0.0186 (5)	0.0118 (4)	−0.0017 (4)	0.0004 (4)	0.0005 (4)
C3	0.0150 (5)	0.0141 (4)	0.0100 (4)	−0.0010 (4)	−0.0005 (4)	−0.0011 (4)
C4	0.0131 (4)	0.0135 (4)	0.0117 (4)	−0.0007 (4)	−0.0003 (4)	−0.0024 (4)
C5	0.0106 (4)	0.0136 (4)	0.0111 (4)	−0.0010 (4)	−0.0003 (4)	−0.0015 (3)
C6	0.0156 (5)	0.0194 (5)	0.0131 (5)	−0.0073 (4)	0.0004 (4)	−0.0013 (4)
C7	0.0117 (4)	0.0210 (5)	0.0137 (4)	−0.0047 (4)	0.0007 (4)	−0.0005 (4)
C8	0.0115 (4)	0.0116 (4)	0.0122 (4)	−0.0015 (4)	0.0016 (4)	−0.0008 (3)
C9	0.0094 (4)	0.0107 (4)	0.0105 (4)	−0.0008 (4)	0.0005 (3)	0.0003 (3)
C10	0.0108 (4)	0.0126 (4)	0.0110 (4)	0.0000 (4)	0.0006 (4)	−0.0003 (4)
C11	0.0119 (4)	0.0129 (4)	0.0116 (4)	−0.0014 (4)	0.0002 (4)	0.0020 (3)
C12	0.0163 (5)	0.0116 (4)	0.0154 (5)	−0.0012 (4)	−0.0020 (4)	0.0004 (4)
C13	0.0122 (4)	0.0101 (4)	0.0110 (4)	−0.0009 (4)	−0.0010 (4)	0.0015 (3)
C14	0.0100 (4)	0.0128 (4)	0.0121 (4)	−0.0005 (4)	0.0008 (4)	0.0002 (3)
C15	0.0142 (5)	0.0162 (5)	0.0133 (4)	−0.0020 (4)	0.0018 (4)	0.0023 (4)
C16	0.0176 (5)	0.0133 (4)	0.0160 (5)	−0.0027 (4)	−0.0023 (4)	0.0038 (4)
C17	0.0130 (4)	0.0124 (4)	0.0126 (4)	0.0005 (4)	−0.0005 (4)	0.0028 (3)
C18	0.0111 (4)	0.0126 (4)	0.0113 (4)	0.0000 (4)	0.0001 (4)	0.0017 (3)
C19	0.0142 (5)	0.0124 (4)	0.0122 (4)	0.0004 (4)	−0.0013 (4)	0.0020 (3)
C20	0.0165 (5)	0.0143 (4)	0.0123 (4)	0.0001 (4)	−0.0011 (4)	−0.0006 (4)
C21	0.0215 (5)	0.0161 (5)	0.0138 (4)	−0.0008 (4)	−0.0046 (4)	0.0021 (4)
C22	0.0194 (5)	0.0156 (5)	0.0120 (4)	−0.0010 (4)	−0.0011 (4)	0.0032 (4)
C23	0.0138 (5)	0.0213 (5)	0.0153 (5)	0.0000 (4)	−0.0023 (4)	−0.0027 (4)
C24	0.0218 (5)	0.0138 (5)	0.0149 (4)	−0.0021 (4)	0.0002 (4)	−0.0037 (4)
C25	0.0190 (5)	0.0161 (5)	0.0162 (5)	0.0052 (4)	0.0020 (4)	0.0001 (4)
C26	0.0235 (6)	0.0110 (4)	0.0162 (5)	0.0004 (4)	0.0024 (4)	0.0007 (4)
C27	0.0136 (5)	0.0188 (5)	0.0171 (5)	0.0047 (4)	0.0013 (4)	−0.0020 (4)
C28	0.0169 (5)	0.0171 (5)	0.0202 (5)	0.0051 (4)	0.0010 (4)	0.0026 (4)
C29	0.0194 (5)	0.0136 (4)	0.0169 (5)	0.0005 (4)	−0.0046 (4)	0.0005 (4)
C30	0.0234 (6)	0.0229 (6)	0.0194 (5)	0.0027 (5)	0.0034 (5)	−0.0034 (4)
C31	0.0146 (5)	0.0167 (5)	0.0144 (5)	0.0019 (4)	−0.0008 (4)	0.0022 (4)
C32	0.0364 (7)	0.0214 (5)	0.0130 (5)	−0.0019 (5)	−0.0025 (5)	0.0018 (4)
C33	0.0185 (5)	0.0212 (5)	0.0359 (7)	−0.0065 (5)	−0.0001 (5)	−0.0029 (5)

### Geometric parameters (Å, °)

**Table:**

O1—C31	1.3475 (13)	C15—H15B	0.9900
O1—C3	1.4603 (12)	C16—C17	1.5476 (16)
O2—C11	1.2033 (15)	C16—H16A	0.9900
O3—C29	1.2025 (15)	C16—H16B	0.9900
O4—C29	1.3389 (15)	C17—C18	1.5059 (14)
O4—C33	1.4444 (15)	C17—C28	1.5332 (16)
O5—C31	1.2039 (15)	C17—C22	1.5455 (15)
O6—C12	1.1953 (14)	C18—C19	1.3299 (15)
O7—C12	1.3735 (13)	C19—C20	1.4939 (15)
O7—C19	1.4024 (13)	C20—C29	1.5293 (17)
C1—C2	1.5342 (15)	C20—C30	1.5400 (17)
C1—C10	1.5416 (15)	C20—C21	1.5554 (16)
C1—H1A	0.9900	C21—C22	1.5304 (17)
C1—H1B	0.9900	C21—H21A	0.9900
C2—C3	1.5177 (16)	C21—H21B	0.9900
C2—H2A	0.9900	C22—H22A	0.9900
C2—H2B	0.9900	C22—H22B	0.9900
C3—C4	1.5428 (16)	C23—H23A	0.9800
C3—H3	1.0000	C23—H23B	0.9800
C4—C24	1.5397 (15)	C23—H23C	0.9800
C4—C23	1.5463 (16)	C24—H24A	0.9800
C4—C5	1.5575 (14)	C24—H24B	0.9800
C5—C6	1.5385 (15)	C24—H24C	0.9800
C5—C10	1.5624 (15)	C25—H25A	0.9800
C5—H5	1.0000	C25—H25B	0.9800
C6—C7	1.5407 (15)	C25—H25C	0.9800
C6—H6A	0.9900	C26—H26A	0.9800
C6—H6B	0.9900	C26—H26B	0.9800
C7—C8	1.5356 (16)	C26—H26C	0.9800
C7—H7A	0.9900	C27—H27A	0.9800
C7—H7B	0.9900	C27—H27B	0.9800
C8—C26	1.5382 (15)	C27—H27C	0.9800
C8—C9	1.5573 (14)	C28—H28A	0.9800
C8—C14	1.6026 (15)	C28—H28B	0.9800
C9—C11	1.5061 (15)	C28—H28C	0.9800
C9—C10	1.5429 (14)	C30—H30A	0.9800
C9—H9	1.0000	C30—H30B	0.9800
C10—C25	1.5448 (15)	C30—H30C	0.9800
C11—C13	1.5582 (15)	C31—C32	1.5044 (16)
C12—C13	1.5478 (15)	C32—H32A	0.9800
C13—C18	1.5006 (14)	C32—H32B	0.9800
C13—C14	1.5910 (15)	C32—H32C	0.9800
C14—C27	1.5366 (15)	C33—H33A	0.9800
C14—C15	1.5538 (15)	C33—H33B	0.9800
C15—C16	1.5332 (16)	C33—H33C	0.9800
C15—H15A	0.9900		
			
C31—O1—C3	117.58 (9)	H16A—C16—H16B	107.7
C29—O4—C33	114.68 (10)	C18—C17—C28	112.02 (9)
C12—O7—C19	107.47 (8)	C18—C17—C22	106.94 (8)
C2—C1—C10	111.77 (9)	C28—C17—C22	110.15 (9)
C2—C1—H1A	109.3	C18—C17—C16	108.17 (9)
C10—C1—H1A	109.3	C28—C17—C16	109.36 (9)
C2—C1—H1B	109.3	C22—C17—C16	110.15 (9)
C10—C1—H1B	109.3	C19—C18—C13	108.86 (9)
H1A—C1—H1B	107.9	C19—C18—C17	123.89 (9)
C3—C2—C1	110.38 (9)	C13—C18—C17	125.65 (9)
C3—C2—H2A	109.6	C18—C19—O7	113.59 (9)
C1—C2—H2A	109.6	C18—C19—C20	128.96 (10)
C3—C2—H2B	109.6	O7—C19—C20	116.88 (9)
C1—C2—H2B	109.6	C19—C20—C29	113.62 (9)
H2A—C2—H2B	108.1	C19—C20—C30	109.68 (10)
O1—C3—C2	110.42 (9)	C29—C20—C30	108.05 (10)
O1—C3—C4	106.08 (8)	C19—C20—C21	107.11 (9)
C2—C3—C4	114.98 (9)	C29—C20—C21	107.30 (9)
O1—C3—H3	108.4	C30—C20—C21	111.08 (10)
C2—C3—H3	108.4	C22—C21—C20	112.09 (10)
C4—C3—H3	108.4	C22—C21—H21A	109.2
C24—C4—C3	111.51 (9)	C20—C21—H21A	109.2
C24—C4—C23	108.66 (9)	C22—C21—H21B	109.2
C3—C4—C23	107.07 (9)	C20—C21—H21B	109.2
C24—C4—C5	113.72 (9)	H21A—C21—H21B	107.9
C3—C4—C5	107.09 (9)	C21—C22—C17	113.28 (9)
C23—C4—C5	108.56 (9)	C21—C22—H22A	108.9
C6—C5—C4	113.51 (9)	C17—C22—H22A	108.9
C6—C5—C10	111.95 (9)	C21—C22—H22B	108.9
C4—C5—C10	116.50 (9)	C17—C22—H22B	108.9
C6—C5—H5	104.4	H22A—C22—H22B	107.7
C4—C5—H5	104.4	C4—C23—H23A	109.5
C10—C5—H5	104.4	C4—C23—H23B	109.5
C5—C6—C7	111.47 (9)	H23A—C23—H23B	109.5
C5—C6—H6A	109.3	C4—C23—H23C	109.5
C7—C6—H6A	109.3	H23A—C23—H23C	109.5
C5—C6—H6B	109.3	H23B—C23—H23C	109.5
C7—C6—H6B	109.3	C4—C24—H24A	109.5
H6A—C6—H6B	108.0	C4—C24—H24B	109.5
C8—C7—C6	111.68 (9)	H24A—C24—H24B	109.5
C8—C7—H7A	109.3	C4—C24—H24C	109.5
C6—C7—H7A	109.3	H24A—C24—H24C	109.5
C8—C7—H7B	109.3	H24B—C24—H24C	109.5
C6—C7—H7B	109.3	C10—C25—H25A	109.5
H7A—C7—H7B	107.9	C10—C25—H25B	109.5
C7—C8—C26	107.97 (9)	H25A—C25—H25B	109.5
C7—C8—C9	107.80 (8)	C10—C25—H25C	109.5
C26—C8—C9	112.47 (9)	H25A—C25—H25C	109.5
C7—C8—C14	115.05 (9)	H25B—C25—H25C	109.5
C26—C8—C14	111.90 (9)	C8—C26—H26A	109.5
C9—C8—C14	101.56 (8)	C8—C26—H26B	109.5
C11—C9—C10	122.43 (9)	H26A—C26—H26B	109.5
C11—C9—C8	100.79 (8)	C8—C26—H26C	109.5
C10—C9—C8	118.71 (9)	H26A—C26—H26C	109.5
C11—C9—H9	104.3	H26B—C26—H26C	109.5
C10—C9—H9	104.3	C14—C27—H27A	109.5
C8—C9—H9	104.3	C14—C27—H27B	109.5
C1—C10—C9	108.78 (8)	H27A—C27—H27B	109.5
C1—C10—C25	107.90 (9)	C14—C27—H27C	109.5
C9—C10—C25	112.43 (9)	H27A—C27—H27C	109.5
C1—C10—C5	108.02 (8)	H27B—C27—H27C	109.5
C9—C10—C5	103.10 (8)	C17—C28—H28A	109.5
C25—C10—C5	116.33 (9)	C17—C28—H28B	109.5
O2—C11—C9	130.77 (10)	H28A—C28—H28B	109.5
O2—C11—C13	124.86 (10)	C17—C28—H28C	109.5
C9—C11—C13	104.34 (9)	H28A—C28—H28C	109.5
O6—C12—O7	120.85 (10)	H28B—C28—H28C	109.5
O6—C12—C13	130.00 (10)	O3—C29—O4	123.70 (12)
O7—C12—C13	109.11 (9)	O3—C29—C20	123.50 (11)
C18—C13—C12	100.80 (9)	O4—C29—C20	112.78 (9)
C18—C13—C11	119.87 (9)	C20—C30—H30A	109.5
C12—C13—C11	104.91 (8)	C20—C30—H30B	109.5
C18—C13—C14	113.05 (8)	H30A—C30—H30B	109.5
C12—C13—C14	114.91 (9)	C20—C30—H30C	109.5
C11—C13—C14	103.52 (8)	H30A—C30—H30C	109.5
C27—C14—C15	106.12 (9)	H30B—C30—H30C	109.5
C27—C14—C13	110.65 (9)	O5—C31—O1	124.17 (10)
C15—C14—C13	108.83 (8)	O5—C31—C32	125.24 (11)
C27—C14—C8	111.26 (9)	O1—C31—C32	110.60 (10)
C15—C14—C8	115.40 (9)	C31—C32—H32A	109.5
C13—C14—C8	104.60 (8)	C31—C32—H32B	109.5
C16—C15—C14	117.84 (9)	H32A—C32—H32B	109.5
C16—C15—H15A	107.8	C31—C32—H32C	109.5
C14—C15—H15A	107.8	H32A—C32—H32C	109.5
C16—C15—H15B	107.8	H32B—C32—H32C	109.5
C14—C15—H15B	107.8	O4—C33—H33A	109.5
H15A—C15—H15B	107.2	O4—C33—H33B	109.5
C15—C16—C17	113.49 (9)	H33A—C33—H33B	109.5
C15—C16—H16A	108.9	O4—C33—H33C	109.5
C17—C16—H16A	108.9	H33A—C33—H33C	109.5
C15—C16—H16B	108.9	H33B—C33—H33C	109.5
C17—C16—H16B	108.9		
			
C10—C1—C2—C3	−58.36 (12)	C11—C13—C14—C27	122.21 (9)
C31—O1—C3—C2	76.94 (12)	C18—C13—C14—C15	9.68 (12)
C31—O1—C3—C4	−157.87 (9)	C12—C13—C14—C15	124.65 (10)
C1—C2—C3—O1	177.46 (9)	C11—C13—C14—C15	−121.55 (9)
C1—C2—C3—C4	57.50 (12)	C18—C13—C14—C8	133.53 (9)
O1—C3—C4—C24	−49.35 (12)	C12—C13—C14—C8	−111.49 (10)
C2—C3—C4—C24	72.98 (12)	C11—C13—C14—C8	2.30 (10)
O1—C3—C4—C23	69.39 (11)	C7—C8—C14—C27	23.18 (12)
C2—C3—C4—C23	−168.28 (9)	C26—C8—C14—C27	146.90 (10)
O1—C3—C4—C5	−174.34 (9)	C9—C8—C14—C27	−92.92 (10)
C2—C3—C4—C5	−52.01 (12)	C7—C8—C14—C15	−97.78 (11)
C24—C4—C5—C6	59.79 (13)	C26—C8—C14—C15	25.93 (13)
C3—C4—C5—C6	−176.57 (9)	C9—C8—C14—C15	146.11 (9)
C23—C4—C5—C6	−61.28 (12)	C7—C8—C14—C13	142.69 (9)
C24—C4—C5—C10	−72.47 (12)	C26—C8—C14—C13	−93.60 (10)
C3—C4—C5—C10	51.18 (12)	C9—C8—C14—C13	26.58 (10)
C23—C4—C5—C10	166.46 (9)	C27—C14—C15—C16	158.17 (10)
C4—C5—C6—C7	163.94 (10)	C13—C14—C15—C16	39.06 (12)
C10—C5—C6—C7	−61.63 (12)	C8—C14—C15—C16	−78.12 (12)
C5—C6—C7—C8	56.45 (13)	C14—C15—C16—C17	−59.65 (13)
C6—C7—C8—C26	71.40 (11)	C15—C16—C17—C18	23.71 (13)
C6—C7—C8—C9	−50.35 (12)	C15—C16—C17—C28	145.97 (10)
C6—C7—C8—C14	−162.83 (9)	C15—C16—C17—C22	−92.85 (11)
C7—C8—C9—C11	−167.72 (9)	C12—C13—C18—C19	−4.12 (12)
C26—C8—C9—C11	73.36 (10)	C11—C13—C18—C19	−118.43 (11)
C14—C8—C9—C11	−46.42 (9)	C14—C13—C18—C19	119.06 (10)
C7—C8—C9—C10	55.67 (12)	C12—C13—C18—C17	−170.08 (10)
C26—C8—C9—C10	−63.26 (12)	C11—C13—C18—C17	75.61 (14)
C14—C8—C9—C10	176.96 (9)	C14—C13—C18—C17	−46.91 (14)
C2—C1—C10—C9	166.68 (9)	C28—C17—C18—C19	103.47 (13)
C2—C1—C10—C25	−71.10 (11)	C22—C17—C18—C19	−17.30 (15)
C2—C1—C10—C5	55.43 (12)	C16—C17—C18—C19	−135.92 (12)
C11—C9—C10—C1	60.76 (12)	C28—C17—C18—C13	−92.58 (13)
C8—C9—C10—C1	−172.31 (9)	C22—C17—C18—C13	146.65 (11)
C11—C9—C10—C25	−58.68 (13)	C16—C17—C18—C13	28.03 (14)
C8—C9—C10—C25	68.25 (12)	C13—C18—C19—O7	4.12 (14)
C11—C9—C10—C5	175.26 (9)	C17—C18—C19—O7	170.38 (10)
C8—C9—C10—C5	−57.82 (11)	C13—C18—C19—C20	−166.81 (11)
C6—C5—C10—C1	172.93 (9)	C17—C18—C19—C20	−0.55 (19)
C4—C5—C10—C1	−54.10 (12)	C12—O7—C19—C18	−2.10 (14)
C6—C5—C10—C9	57.89 (11)	C12—O7—C19—C20	170.00 (10)
C4—C5—C10—C9	−169.14 (9)	C18—C19—C20—C29	−129.02 (13)
C6—C5—C10—C25	−65.62 (11)	O7—C19—C20—C29	60.31 (14)
C4—C5—C10—C25	67.34 (12)	C18—C19—C20—C30	109.94 (14)
C10—C9—C11—O2	5.92 (19)	O7—C19—C20—C30	−60.74 (13)
C8—C9—C11—O2	−128.53 (13)	C18—C19—C20—C21	−10.71 (17)
C10—C9—C11—C13	−176.09 (9)	O7—C19—C20—C21	178.61 (10)
C8—C9—C11—C13	49.45 (9)	C19—C20—C21—C22	40.24 (13)
C19—O7—C12—O6	177.07 (12)	C29—C20—C21—C22	162.58 (10)
C19—O7—C12—C13	−0.79 (12)	C30—C20—C21—C22	−79.52 (12)
O6—C12—C13—C18	−174.68 (13)	C20—C21—C22—C17	−63.10 (13)
O7—C12—C13—C18	2.92 (12)	C18—C17—C22—C21	47.71 (13)
O6—C12—C13—C11	−49.55 (17)	C28—C17—C22—C21	−74.24 (12)
O7—C12—C13—C11	128.06 (9)	C16—C17—C22—C21	165.04 (10)
O6—C12—C13—C14	63.44 (17)	C33—O4—C29—O3	1.44 (18)
O7—C12—C13—C14	−118.96 (10)	C33—O4—C29—C20	179.73 (10)
O2—C11—C13—C18	19.22 (17)	C19—C20—C29—O3	−162.49 (12)
C9—C11—C13—C18	−158.92 (9)	C30—C20—C29—O3	−40.54 (16)
O2—C11—C13—C12	−92.91 (14)	C21—C20—C29—O3	79.32 (14)
C9—C11—C13—C12	88.95 (10)	C19—C20—C29—O4	19.21 (14)
O2—C11—C13—C14	146.27 (12)	C30—C20—C29—O4	141.16 (10)
C9—C11—C13—C14	−31.87 (10)	C21—C20—C29—O4	−98.98 (11)
C18—C13—C14—C27	−106.56 (10)	C3—O1—C31—O5	−3.23 (18)
C12—C13—C14—C27	8.41 (12)	C3—O1—C31—C32	176.93 (10)

### Hydrogen-bond geometry (Å, °)

**Table:**

*D*—H···*A*	*D*—H	H···*A*	*D*···*A*	*D*—H···*A*
C2—H2B···O5	0.99	2.58	3.1121 (14)	114
C9—H9···O6	1.00	2.43	3.1601 (13)	129
C23—H23A···O1	0.98	2.49	2.9084 (15)	106
C25—H25A···O2	0.98	2.60	3.2898 (16)	127
C27—H27B···O6	0.98	2.49	3.0454 (16)	116
C1—H1A···O5^i^	0.99	2.63	3.4839 (15)	145
C5—H5···O5^ii^	1.00	2.64	3.5313 (14)	149
C7—H7B···O2^iii^	0.99	2.59	3.1743 (15)	118
C7—H7B···O5^ii^	0.99	2.70	3.5497 (15)	145

Symmetry codes: (i) *x*−1/2, −*y*+1/2, −*z*+1; (ii) *x*+1/2, −*y*+1/2, −*z*+1; (iii) *x*+1, *y*, *z*.

## References

[bb1] Amer, H., Mereiter, K., Stanetty, C., Hofinger, A., Czollner, L., Beseda, I., Jordis, U., Kueenburg, B., Classen-Houben, D. & Kosma, P. (2010). *Tetrahedron*, **66**, 4390–4402.

[bb2] Asl, M. N. & Hosseinzadeh, H. (2008). *Phytother. Res.* **22**, 709–724. 10.1002/ptr.2362 PMC7167813 18446848

[bb3] Baran, J. S., Langford, D. D., Liang, C. & Pitzele, B. S. (1974). *J. Med. Chem.* **17**, 184–191. 10.1021/jm00248a008 4203364

[bb4] Beseda, I., Czollner, L., Shah, P. S., Khunt, R., Gaware, R., Kosma, P., Stanetty, C., del Ruiz-Ruiz, M. C., Amer, H., Mereiter, K., Da Cunha, T., Odermatt, A., Classen-Houben, D. & Jordis, U. (2010). *Bioorg. Med. Chem.* **18**, 433–454. 10.1016/j.bmc.2009.10.036 19914836

[bb5] Brownlie, G. & Spring, F. S. (1956). *J. Chem. Soc.* pp. 1949–1953.

[bb6] Bruker (2008). *APEX2*, *SAINT* and *SADABS* Bruker AXS Inc., Madison, Wisconsin, USA.

[bb7] Classen-Houben, D., Schuster, D., Da Cunha, T., Odermatt, A., Wolber, G., Jordis, U. & Kueenburg, B. (2009). *J. Steroid Biochem. Mol. Biol.* **113**, 248–252. 10.1016/j.jsbmb.2009.01.009 19429429

[bb8] Jeger, O., Norymberski, J. & Ruzicka, L. (1944). *Helv. Chim. Acta*, **27**, 1532–1543.

[bb9] Kitagawa, I. (2002). *Pure Appl. Chem.* **74**, 1189–1198.

[bb10] Macrae, C. F., Edgington, P. R., McCabe, P., Pidcock, E., Shields, G. P., Taylor, R., Towler, M. & van de Streek, J. (2006). *J. Appl. Cryst.* **39**, 453–457.

[bb11] Sheldrick, G. M. (2008). *Acta Cryst.* A**64**, 112–122. 10.1107/S0108767307043930 18156677

[bb12] Spek, A. L. (2009). *Acta Cryst.* D**65**, 148–155. 10.1107/S090744490804362X PMC2631630 19171970

[bb13] Westrip, S. P. (2010). *J. Appl. Cryst.* **43**, 920–925.

